# Host plant driven transcriptome plasticity in the salivary glands of the cabbage looper (*Trichoplusia ni*)

**DOI:** 10.1371/journal.pone.0182636

**Published:** 2017-08-08

**Authors:** Loren J. Rivera-Vega, David A. Galbraith, Christina M. Grozinger, Gary W. Felton

**Affiliations:** Department of Entomology, Pennsylvania State University, University Park, PA, United States of America; USDA Agricultural Research Service, UNITED STATES

## Abstract

Generalist herbivores feed on a wide array of plants and need to adapt to varying host qualities and defenses. One of the first insect derived secretions to come in contact with the plant is the saliva. Insect saliva is potentially involved in both the pre-digestion of the host plant as well as induction/suppression of plant defenses, yet how the salivary glands respond to changes in host plant at the transcriptional level is largely unknown. The objective of this study was to determine how the labial salivary gland transcriptome varies according to the host plant on which the insect is feeding. In order to determine this, cabbage looper (*Trichoplusia ni*) larvae were reared on cabbage, tomato, and pinto bean artificial diet. Labial glands were dissected from fifth instar larvae and used to extract RNA for RNASeq analysis. Assembly of the resulting sequencing reads resulted in a transcriptome library for *T*. *ni* salivary glands consisting of 14,037 expressed genes. Feeding on different host plant diets resulted in substantial remodeling of the gland transcriptomes, with 4,501 transcripts significantly differentially expressed across the three treatment groups. Gene expression profiles were most similar between cabbage and artificial diet, which corresponded to the two diets on which larvae perform best. Expression of several transcripts involved in detoxification processes were differentially expressed, and transcripts involved in the spliceosome pathway were significantly downregulated in tomato-reared larvae. Overall, this study demonstrates that the transcriptomes of the salivary glands of the cabbage looper are strongly responsive to diet. It also provides a foundation for future functional studies that can help us understand the role of saliva of chewing insects in plant-herbivore interactions.

## Introduction

Generalist insects feed on a wide array of plants and thus are exposed to a diversity of plant nutritional qualities, as well as different secondary metabolites [1]. Generalist insects have evolved mechanisms to cope with plant defenses, including behavioral avoidance of resistant plants, metabolism of toxic compounds, and even suppression of the induced defenses through the release of suppressive effectors during feeding [2–5]. Mechanisms by which generalist insects cope with the defensive compounds of different plants have been evaluated. Most of these studies have focused on midgut responses due to its known role in digestion and detoxification [6,7]. However, insects interact with host plant tissue well before digestion is initiated. In fact, the first insect secretion that interacts with plant tissue during feeding is the insect's saliva[8]. In caterpillars, saliva arises from the labial and mandibular glands [9]. Labial saliva is released through the spinneret, which is external to the oral cavity and thus allows saliva to be released on the plant before ingestion. Saliva from chewing insects may play a role in digestion, detoxification, immunity and suppression of plant defenses [9,10] and may represent the insect’s first line of defense against plant secondary compounds.

Plants, on the other hand, are able to differentiate between mechanical wounding and herbivory damage [11], which indicates the recognition of insect specific molecules by the plant. Insect oral secretions (mix of regurgitant and saliva) have been found to induce plant defenses [12–14]. Some of the elicitors of plant defense identified in caterpillar oral secretions include: fatty-acid amino acid conjugates[15], a beta-glucosidase[16], inceptin[17], and a porin-like protein[18]. However, the role of saliva specifically has not been thoroughly studied. To date, salivary molecules from chewing insects known to play a role in plant-insect interactions include: ATP-utilizing enzymes[4] and glucose oxidase[3].

Glucose oxidase is the most well studied salivary enzyme in caterpillars. Glucose oxidase produced by the salivary glands of *Helicoverpa zea* caterpillars has been found to suppress plant defenses in tobacco [3]. Glucose oxidase synthesis and secretion varies according to host plant or diet as well as the dietary carbon to protein ratio [19–21]. This provides some evidence of plasticity in the salivary glands of chewing insects in response to different diets and host plants. However, the effect of host plants in the overall salivary composition of chewing insects requires further study. Plasticity is the ability of an organism to express different phenotypes depending on the environment [22, 23]. These changes can be either biotic or abiotic: host plant, temperature, light conditions, presence of predators, etc. The environmental changes can be continuous such as in the case of temperature or discrete as in the case of different host plants for a generalist insect [24].

We chose the system of cabbage looper (*Trichoplusia ni*) feeding on cabbage (*Brassica oleracea* var capitata) and tomato (*Solanum lycopersicum*) to study the overall transcriptomic changes that occur in the salivary glands of a generalist, chewing insect when feeding on different host plant species. Cabbage looper is a generalist herbivore from the lepidopteran family Noctuidae. The larvae feed on more than 50 plant species from several families [25]. Although it preferentially feeds on plants of the Brassicaceae family, it is also considered a pest for plants from the Solanaceae, Convolvulaceae, Cucurbitaceae families, among others. Because of its broad host range, the cabbage looper is exposed to a wide range of defensive compounds including general defenses such as phenolics, alkaloids and terpenes, protease inhibitors, polyphenol oxidases and other defensive proteins, as well as the more specific defenses found in Brassicaceae such as glucosinolates [26,27]. It is critical for the larvae to be able to detoxify each of these defenses, which may involve different mechanisms. Since continuously expressing its full arsenal of defensive responses would presumably be energetically costly, we hypothesize the responses of the cabbage looper larvae must be plastic in order to allow it to utilize a broad range of host plant species. A recent study has shown remodeling of the midgut transcriptome in the cabbage looper when feeding on different hosts [28]; however, whether such plasticity is also present in the salivary glands of this insect is still unknown. We hypothesize the salivary glands of the cabbage looper are plastic and respond to different host plants at the transcriptomic level. To test this hypothesis, we reared the larvae on tomato, cabbage, and artificial diet (control) and then analyzed the overall gene expression in the salivary glands using RNASeq technology.

## Materials and methods

### Plants and insects

Cabbage looper (*Trichoplusia ni*) eggs were purchased from BioServ Inc (Flemington, NJ). Larvae were reared entirely on two host plants: tomato (*Solanum lycopersicum* var. Better Boy) and cabbage (*Brassica oleracea* var capitata ‘Platinum Dynasty’), and pinto-bean artificial diet [29]. Whole plants were used instead of clippings and food was never limited to the insects. Colonies were kept at 23°C in 16:8 Light:Dark conditions. Tomato and cabbage plants were grown in the greenhouse under a 16:8 L:D cycles and fertilized as needed. Days to pupation for 60 caterpillars reared on each treatment were measured.

### RNA isolation and sequencing

Fifth instar larvae were allowed to feed for at least 1 day after molt, and then collected from the different treatments (tomato, cabbage and artificial diet). Samples were always collected early in the afternoon from larvae actively feeding. Salivary glands were dissected in chilled PBS buffer and immediately placed in liquid nitrogen and stored at -80°C until further processing. Each sample contained a pool of 10 pairs of salivary glands. Total RNA was extracted using the QIAGEN RNeasy kit (Valencia, CA) following the manufacturer’s protocol. Sample quality and quantity were assessed and validated using the Agilent bioanalyzer (Agilent Technologies, Inc; Santa Clara, CA). Samples were submitted to the Genomics Core Facility at The Pennsylvania State University to be sequenced using the Illumina HiSeq 2500 (San Diego, CA). Three biological replicates per treatment were sequenced for a total of nine samples. A barcoded library was made from each sample using the Illumina TruSeq Stranded mRNA Library Prep Kit (#RS-122-2101) according to the manufacturer's protocol. The concentration of each library was determined by RTqPCR and an equimolar pool of the libraries was sequenced on the Illumina HiSeq 2500 in Rapid Run Mode using 100 nt single read.

### Transcriptome analysis

Transcriptome sequencing reads were processed with Trimmomatic v0.32 [30], removing low quality reads, adaptor sequences, and reads with more than 5% unknown bases. A set of non-redundant transcripts was generated *de novo* using Trinity v2.1.0 using the default parameters [31]. The processed reads were aligned to this reference transcriptome using Tophat v2.0.10 [32]. Read counts for each transcript were imported into R v3.0.2 for further analyses. Transcripts with low read counts (<10 across all samples) were removed from further analyses. The data was normalized using a trimmed mean of M-values (TMM) method [33] and tested for differential expression using a generalized linear model in EdgeR v3.4.2 [33]. Transcripts were considered to be significantly differentially expressed when FDR < 0.05. Pairwise comparisons of the three treatments were performed to identify the effects of the three different diets on salivary gland gene expression. The generated transcripts were annotated using a reciprocal best hit BLAST [34] approach to identify *Bombyx mori* orthologs. These orthologs were then used for a gene ontology (GO) analysis using DAVID v6.7 [35]. Functional categories were then clustered by parent GO terms using REVIGO [36]. The same samples used for RNA-Seq were later used for validations using real time quantitative PCR. Sequence reads and assembled transcripts generated from this study have been deposited in the Gene Expression Omnibus [37] with accession number GSE101549.

### Real time quantitative PCR

Real time quantitative PCR (RTqPCR) was used to validate results from the bioinformatic analysis. One μg of RNA was used to synthesize cDNA using the High Capacity cDNA Reverse Transcription kit following manufacturer’s protocol (Applied Biosystems, Inc; Grand Island, NY). Primers were designed for 9 of the differentially expressed genes along with 4 other genes involved in detoxification (S1 Table). All reactions were done using Power SYBR Green PCR Master Mix and ran on a 7500 Fast Real-Time PCR System (Applied Biosystems, Inc; Grand Island, NY). Relative expression for each gene was quantified using the ΔΔCt method [38], and normalized using GAPDH. Actin was also assessed as potential reference gene. GAPDH was the most stable gene across samples. Standard curves using serial dilutions and melting curves were performed to calculate primer efficiency (E = 10^^(-1/slope)^-1) and confirm the presence of single amplicon.

### Role of saliva in detoxification

For validation of the role of salivary glands in detoxification, third instar larvae were transferred to tomato plants from *def*-1 (*Defenseless*-1) mutants and Castlemart variety–background for *def*-1 [39]. Def-1 mutants are affected in the octadecanoid pathway and because of this, they do not respond to insect damage. Plants were grown under same conditions as previously described. Salivary glands were dissected from fifth instar larvae allowed to feed for at least 24 h post molting and used for RTqPCR as previously described. Genes analyzed were: catalase, glutathione-S-transferase, protease, proteinase inhibitor, cytochrome P450, and UDP-glycosyl transferase (S1 Table).

### Statistics

Differences in days to pupation were determined using ANOVA followed by a separation of means using a Tukey post-hoc test at p<0.05. Differences in gene expression using RTqPCR were analyzed using the nonparametric test Mann-Whitney U test. For statistics of transcriptome analyses refer to Transcriptome section described above. Heatmaps were made using the heatmap3 package from R. All statistics were done using R software.

## Results and discussion

Generalist insects feed on a wide array of plants and because of this, they are exposed to a diversity of primary and secondary compounds. Understanding this ability to establish on so many different plants has long been the aim of chemical ecologists. Both behavioral and physiological adaptations have been reported in cabbage loopers that could allow them to cope with the myriad of plant defenses to which they are exposed [2,40]. For example, changes in midgut transcriptome have been reported in the cabbage looper when feeding on defended and undefended hosts [28]. This plasticity might also be present in other tissues in the insect. Saliva is one of the first secretions to come in contact with the plant during feeding and so it could be playing an important role in an insect’s ability to successfully feed on a host. The main goal of this study was to determine the overall transcriptomic changes that occur in the salivary glands of the generalist cabbage looper when it feeds on different hosts.

We established a transcriptome library for the salivary glands of the cabbage looper. We sequenced three biological replicates for three treatments (cabbage fed, tomato fed, and artificial diet fed) for a total of 9 samples. The number of reads generated from each sample ranged from 25,714,877 to 28,381,856. The Trinity assembly generated 38,082 transcripts that corresponded to 30,082 'Trinity genes' (a collection of related transcripts–S2 Table). These were clustered into 14,037 components, out of which, 7,913 corresponded to *Bombyx mori* orthologs based on a reciprocal best-hit blast approach. (S3 Table).

### Cabbage looper grows at different rates on different host plants

**Fig 1:**
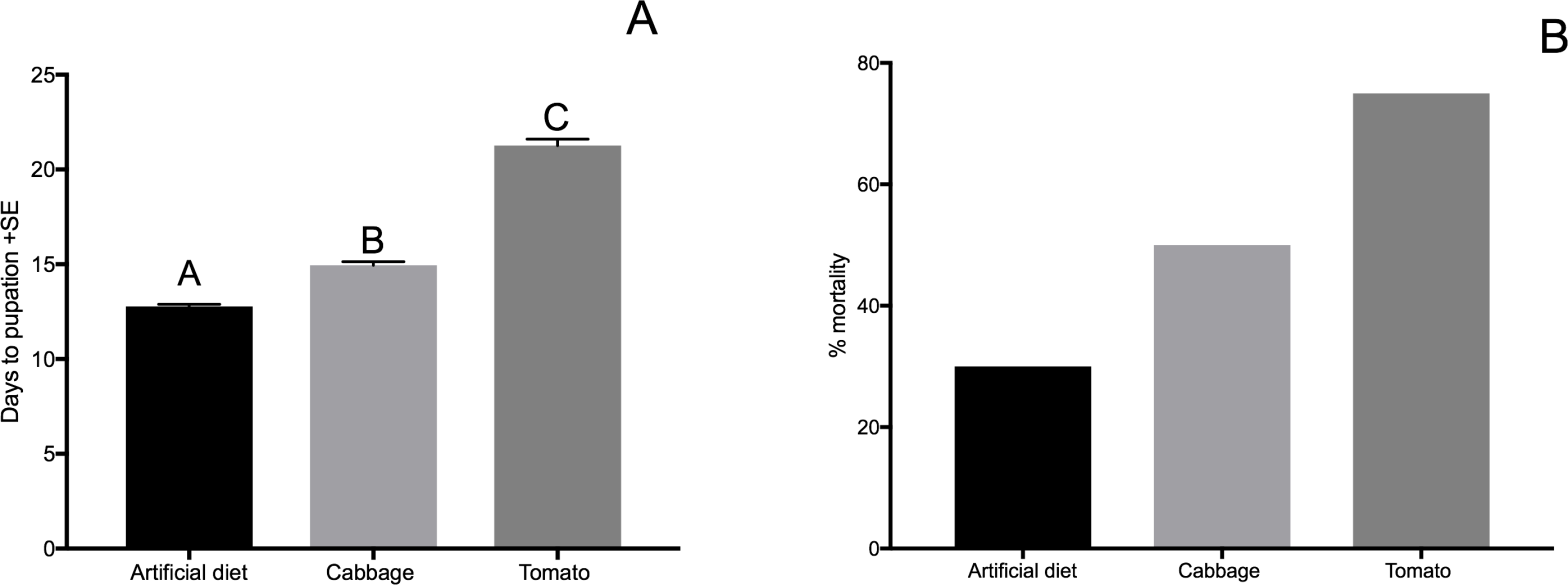
Effect of different diets on the growth of cabbage looper (*Trichoplusia ni*). **A**. Days to pupation of cabbage looper reared on three treatments–cabbage, tomato, and pinto bean artificial diet. An ANOVA was done followed by Tukey post hoc test at p<0.05 to determine statistical differences. F(2,178) = 465, pvalue<0.0001. **B.** Percentage mortality of larvae on each diet.

Plant quality can be measured several ways. A common way is by measuring carbon or carbohydrates, nitrogen or protein, and secondary compounds [41]. Though it is normally assumed that higher nitrogen means better quality, the origin of this nitrogen is not specific. This means that it could come from a source that is not beneficial or available for the insect. Furthermore, the effect of protein and secondary compounds is context dependent [42]. For example, some plant defenses might have a toxic effect on insects in the presence of higher protein quality [43], which is counterintuitive. In this respect, a better measurement is to actually measure the effect the plant has on the insect i.e. growth, fecundity, mortality, etc. Here, we measured how long it took larvae to reach pupation along with mortality when feeding on each host plant (Fig 1).

The cabbage looper, even though considered a generalist, commonly grows faster when feeding on plants from the Brassicaceae family. However, it is still able to complete its cycle in plants from other families. There is evidence that this difference in growth is in part due to the different secondary chemistries of the hosts [28]. However, there may also be nutritional differences mediating these responses: Herde and Howe (2014) demonstrated reduced growth of caterpillars on mutant tomato plants versus mutant Arabidopsis plants, even though the plants were mutated to reduce defense responses, thus indicating an effect due to something other than induced defenses.

Cabbage loopers were reared on cabbage, tomato and artificial diet. Treatments had a significant effect on the number of days required for larvae to reach pupation. Larvae that were reared on the artificial diet and cabbage reached pupation in approximately 13 and 15 days, respectively; whereas, larvae reared on tomato required an average of 23 days to reach pupation (Fig 1A). Also, a higher percentage of larvae died when feeding on tomato compared to cabbage and artificial diet (Fig 1B). Because of the differential growth of the caterpillars and percentage mortality on cabbage versus tomato, we refer to cabbage as a "high quality host" and tomato as a "poor quality host".

### Transcriptomes of cabbage looper salivary glands are extensively remodeled according to host plant species

**Fig 2:**
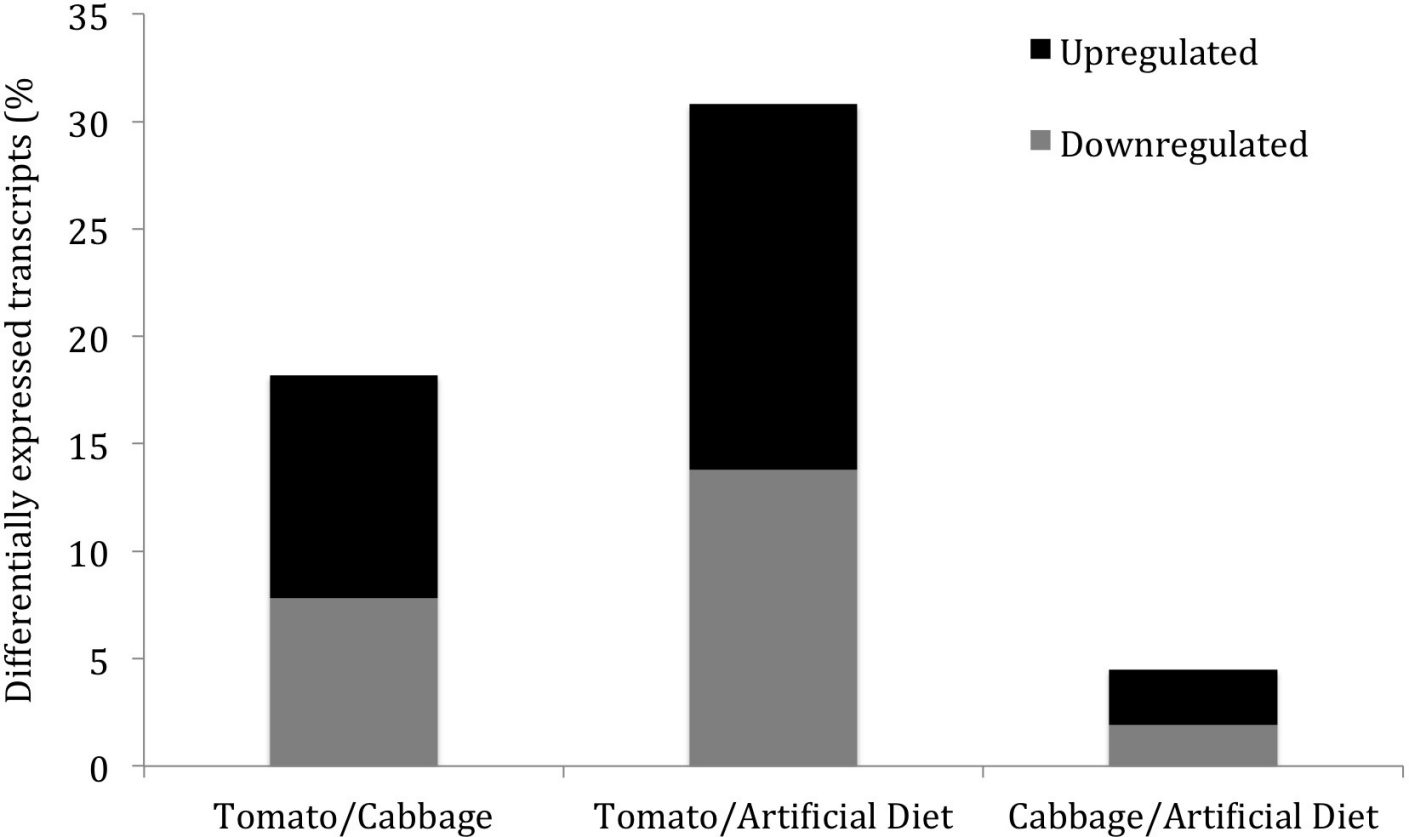
Percentage of differentially expressed genes from total transcriptome library for three comparisons–Tomato vs. Cabbage, Tomato vs. Artificial diet, and Cabbage vs. Artificial diet. Black indicates upregulated genes and grey indicate downregulated genes for each comparison.

When comparing gene expression of salivary glands from larvae reared in cabbage (high quality host) against those in artificial diet (control), only 630 (4% of the total transcripts) transcripts were significantly differentially expressed, with 366 transcripts being upregulated and 264 downregulated. A much larger proportion of transcripts were significantly differentially expressed in the glands of larvae reared on tomato (low quality host) compared to larvae reared on artificial diet, where 4,318 transcripts (representing 31% of the total transcripts) were differentially expressed, with 2,386 being upregulated and 1,932 downregulated. Finally, in the cabbage to tomato comparison, 1,100 transcripts were upregulated on cabbage fed larvae and 1,466 downregulated for a total of 2566 differentially expressed transcripts representing 18% of the total transcripts (Fig 2). Nine genes were chosen for validations of RNASeq results using RTqPCR. Out of the 27 comparisons made (3 per gene), only 5 did not correlate between the RNASeq and real time qPCR results (S1 Fig).

Clustering analysis of all the genes revealed that the transcription profiles of salivary glands of larvae fed on cabbage and artificial diet were similar to each other, while the profile of the glands of tomato-fed larvae were extensively different (S2 Fig). The greatest changes were observed in the salivary glands of tomato-fed insects, which were also the insects that had the slowest growth. This plasticity in a generalist insect is consistent with a previous study that observed changes in the transcriptome of the generalist two-spotted spidermite (*Tetranychus urticae*) when it fed on tomato plants. In this study, a total of 1,275 genes were differentially expressed [44]. In contrast, the salivary glands of the tobacco hornworm (*Manduca sexta*), a specialist of Solanaceae did not exhibit such a dramatic change in gene expression when exposed to different host plants [45].

### Spliceosome pathway genes show significant downregulation in larvae reared on tomato

A Kyoto Encyclopedia of Genes and Genomes (KEGG) pathway analysis identified the spliceosome pathway as significantly downregulated (p-value: 1.42X10^-7^) in larvae reared on tomato versus artificial diet. The spliceosome is the ribonucleoprotein complex involved in splicing or intron removal of pre-mRNAs, and is involved in both constitutive and alternative splicing of mRNAs [46]. There is little information about the mechanisms and factors that regulate alternative splicing [47], therefore it is difficult to determine the biological significance of differential regulation of this pathway. Also, even though a downregulation of this pathway is observed, transcription change might not translate to protein change. However, we can make at least three hypotheses of what a downregulation of the spliceosome could mean in this system. First, the stress of feeding on tomato could be causing the insect to downregulate transcription in general, including the splicing machinery. In this case, we would expect the proportion of downregulated genes to be higher than the upregulated ones. However, we did not observe this (Fig 2). Second, the downregulation of the spliceosome alone could be a response to the stress of feeding on tomato and could be impacting the protein synthesis machinery in general. Finally, the downregulation of this pathway could signify that the salivary glands are switching to different variants of some proteins. Alternative splicing allows eukaryotic organisms to increase their proteome without having to remodel their genome extensively, thus providing an efficient plasticity mechanism [48]. In plants, it has been shown that different stresses have a dramatic effect on alternative splicing, including the spliceosome itself [49]. Insects have been shown to have even higher levels of alternative splicing than plants [50] and thus insects could be utilizing a similar mechanism to cope with stresses due to plant defenses. It remains to be determined whether the plant’s defensive mechanism influences this change in the splicing machinery, or if it is an adaptation of the insect. Factors that are involved in suppression of alternative splicing regulation were identified in our transcriptome [47], including: polypyrimidine tract binding protein (PTB/HNRNP1), heterogeneous nuclear ribonucleoprotein variant A1 (hnRNPA1), sex lethal variant L (SXL) and U2 auxiliary factor 35 kDA (U2AF35). The availability of this library could provide a useful resource for studying different factors that affect alternative splicing and/or the role of alternative splicing in coping with stress in a non-model organism.

### Candidate genes may play a role in mediating cabbage looper caterpillar interaction with different host plant species

#### Digestion

**Fig 3:**
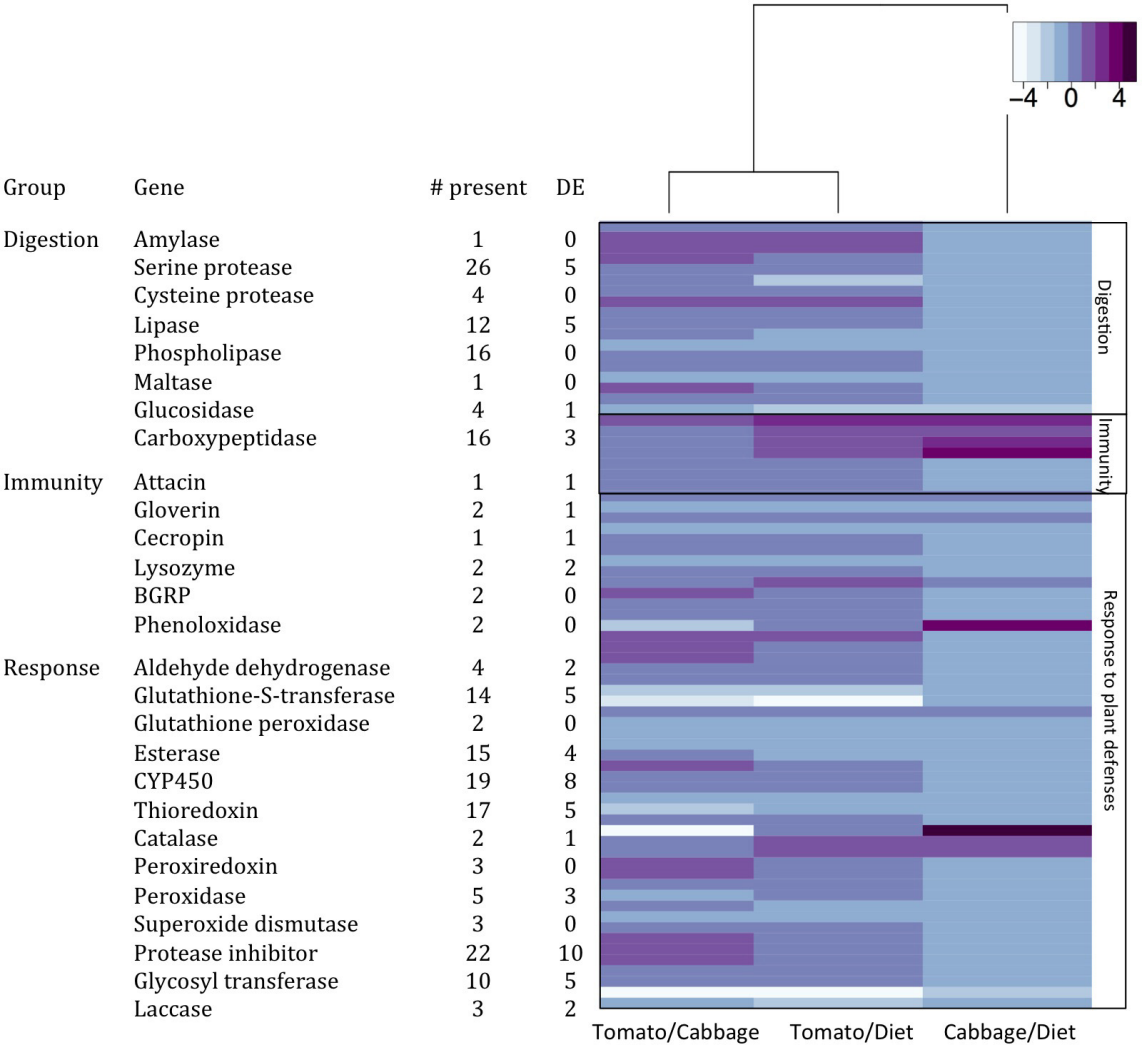
Genes of interest and their differential expression (DE). **A. List of genes of interest identified in the transcriptome of cabbage looper (*Trichoplusia ni*) salivary glands.** # present–indicates number of transcripts identified. DE indicates how many of the identified transcripts were differentially expressed. B. Heatmap of genes of interest in three comparisons–Tomato vs. Cabbage, Tomato vs. Artificial Diet, and Cabbage vs. Artificial Diet. Darker colors (purple) indicate upregulation in each comparison, light blue indicates no significant differential expression and white indicates downregulation. Only transcripts differentially expressed in at least one of the comparisons were included.

Saliva is involved in digestion in almost every organism that produces it. Because caterpillars release their saliva through an extra-oral structure (spinneret), this could mean that digestion begins before ingestion. The presence of digestive enzymes in the saliva of insects has been widely studied in aphids and other sucking insects [51,52]. Digestive enzymes have also been identified in the salivary glands of a handful of chewing insects (*Helicoverpa armigera*, *Helicoverpa zea*, *Manduca sexta*)[10,45,53] but their role and differential expression has not been confirmed. We identified numerous transcripts regularly associated with digestion, including alpha-amylases, lipases, proteases, and glucosidases. Many of these transcripts were differentially expressed including serine proteases, carboxypeptidases, and lipases (Fig 3). Most of these were upregulated in the salivary glands of insects reared on tomato plants. Herde and Howe (2014) observed upregulation of serine proteases and downregulation of lipases in the midgut when cabbage looper caterpillars fed on tomato. In the midgut, several digestive enzymes are secreted through vesicles[54,55]. This is probably also the case in the salivary glands. These compounds could be functioning to digest the plant before ingestion but they could also be helping the already present digestive enzymes in the midgut thus allowing for a faster and more efficient digestion. A similar effect has been previously observed in the American cockroach (*Periplaneta americana*): the amylase present in the midgut is produced in the salivary glands and mixed with the food to aid in digestion occurring in the midgut [56]. The presence of digestive transcripts in the salivary glands of the cabbage looper provides more evidence that caterpillars could be depositing these molecules to aide in pre-ingestive or extra-oral digestion. This would be consistent with previous studies in predaceous arthropods [57], where predators will deliver saliva into the prey in order to aid in digestion prior to ingesting it. Alternatively, the proteases may be involved in adaptation to dietary proteinase inhibitors [58,59] and not just in the breakdown of macromolecules.

#### Immunity

Host plants are known to affect an insect’s immune response [60,61]. For example, in the autumnal moth (*Epirrita automnata*), differences in encapsulation rate were observed depending on food quality [62]. Because of this, we also screened for transcripts involved in immunity (i.e., attacin, cecropin, BGRP, gloverin, lysozymes). Almost 40% of these transcripts (4 out of 11) were differentially expressed (Fig 3). All of these were upregulated when insects were reared on plants compared to artificial diet.

Another possibility is that this differential expression could be due to the microorganisms present on the plant surface. The artificial diet contains antimicrobial compounds, which means it should contain fewer microbes. Differences in immunity related genes in the salivary glands of *Manduca sexta* feeding on different host plants has also been observed [45]. The authors of this study suggest that these differences were probably due to different microbial communities associated with different host plants. However, this is yet to be tested. The induction of these immune genes in the saliva of insects feeding on plant provides more evidence that it is important to consider the microbiome associated with each plant, as well as the primary and secondary chemistry when studying plant-insect interactions.

#### Response to plant defenses

**Fig 4:**
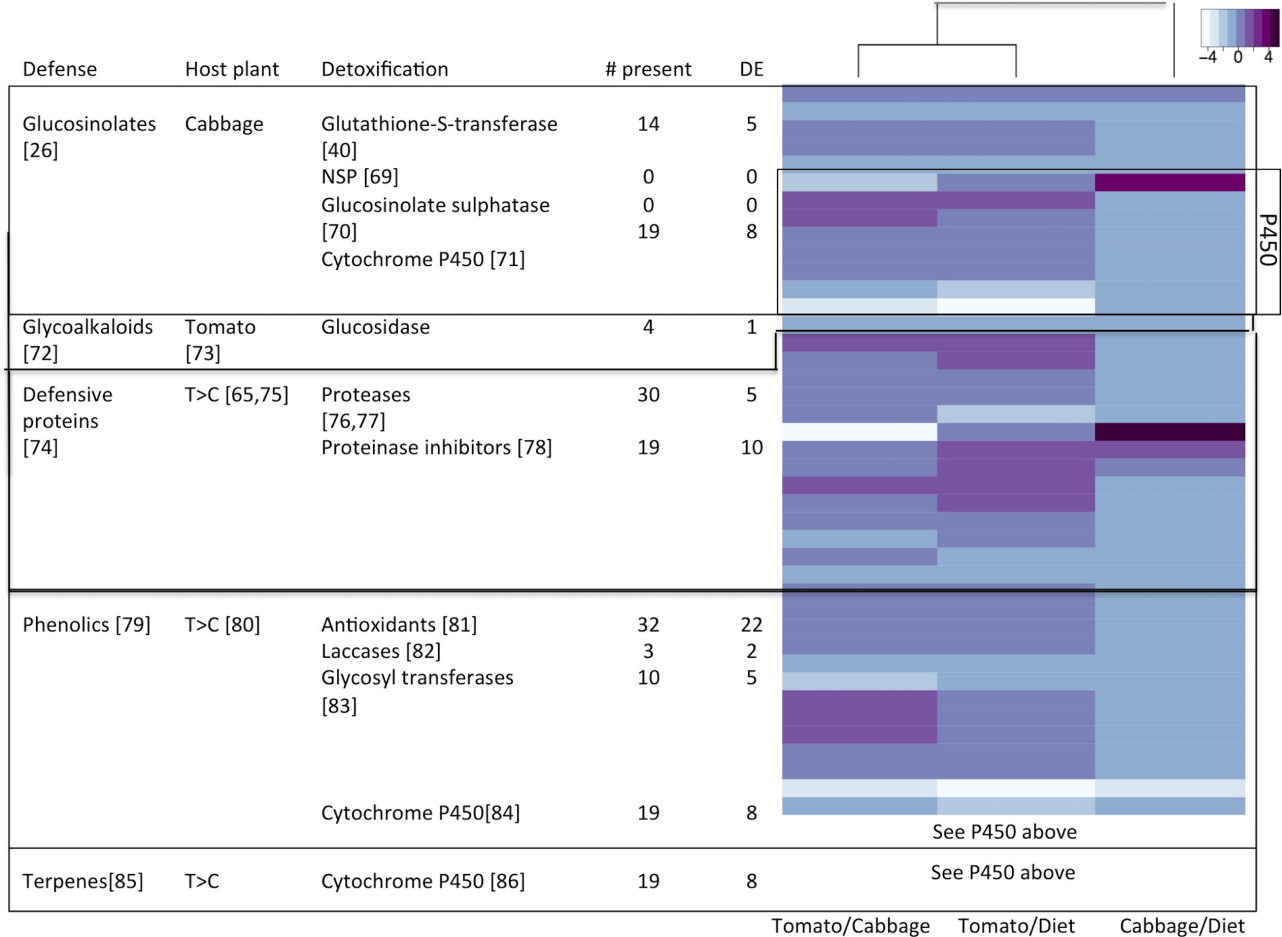
Differential expression (DE) of genes involved in the detoxification of cabbage and tomato specific defenses. A. List of defensive compounds associated with different hosts (cabbage and tomato) and insect-related detoxification genes. B. Heatmap of detoxification genes in three comparisons–Tomato vs. Cabbage, Tomato vs. Artificial Diet, and Cabbage vs. Artificial Diet. Darker colors (purple) indicate upregulation in each comparison, light blue indicates no significant differential expression and white indicates downregulation. Only transcripts differentially expressed in at least one of the comparisons were included [26,40,65,69–86].

We also identified a several detoxification/antioxidant transcripts. These could be having an active role in the detoxification of plant secondary compounds and mitigating oxidative stress. Moreover, some of these proteins could be suppressing or manipulating the signals in the plant to avoid recognition. It has been hypothesized that the evolution of dietary generalism in insects came with the trade off of reduced ability to respond to the defensive compounds of different plant families [63]. However, plasticity in expression of the genes involved in detoxification of such compounds could allow a generalist insect to still feed on multiple chemically divergent plant species. Cabbage and tomato rely on different defensive strategies, utilizing different molecules to ward off herbivores. Cabbage, as part of the Brassicaceae family, contains glucosinolates and to some extent proteinase inhibitors. Glucosinolates are hydrolyzed into isothiocyanates, which are toxic and interact with amino groups of proteins and cleave disulfide bonds [64]. Proteinase inhibitors reduce the availability of sulphur-containing amino acids and cause the hyperproduction of trypsin [65]. In contrast, tomato contains high amounts of phenolics and proteinase inhibitors [64,66,67]. Phenolics affect the insect by forming quinones which react with nucleophilic side chains of amino acids making the amino acids unavailable [68]. Differential expression of genes potentially involved in the detoxification of these compounds was observed (Fig 4).

Detoxification of plant defensive compounds is typically divided in two phases. In Phase I, toxic compounds are oxidized, hydrolyzed, or reduced; the resulting compounds are normally nonpolar and cannot be excreted directly. In Phase II the compounds from Phase I are conjugated with other compounds such as sugars, sulfate, phosphate, amino acids or glutathione, and then excreted [87]. Among the proteins involved in detoxification are cytochrome P450s, glutathione-S-transferases, glycosyl transferases, aldehyde reductases, esterases, and others. We identified 82 genes involved in detoxification processes, with 42 of them being differentially expressed. Most of these were significantly differentially regulated in the tomato vs. artificial diet comparison, where 39 of the 42 differentially expressed genes were present (25 upregulated and 14 downregulated). Protease inhibitors, cytochrome P450s, thioredoxins and glutathione-S-transferases were the most prominent detoxification genes that were differentially expressed in response to feeding on plants.

As a defense mechanism against herbivores, some plants induce protease inhibitors that can affect digestibility in the insect, and also release proteases that can cause damage to the insect itself. For example, maize produces a cysteine protease that damages the peritrophic membrane of caterpillars [88]. A way in which insects could counteract this defense is by producing inhibitors that target plant proteases. In fact, it has been shown that the cabbage looper induces cysteine protease inhibitors to counteract cysteine proteases that might degrade their peritrophic membrane[78]. These results point towards an active role of caterpillar saliva in response to plant induced defenses.

Because insects have an open circulatory system, it is expected that all tissues will have a certain level of detoxification capability in order to overcome harmful compounds found in the hemolymph as well as to detoxify compounds produced during regular cellular metabolism. However, some of the compounds identified here as potentially being involved in response to plant defenses (i.e. proteases, protease inhibitors, carboxylesterases, and others) have been identified in the secreted saliva of *Helicoverpa zea* larvae and *Heliconius melpomene* adults [53,89,90]; thus, pointing towards a potential role in extra-oral detoxification. By releasing detoxifying enzymes prior to ingestion, the cabbage looper could both reduce the pressure of detoxification in the midgut and the exposure of the digestive tract to potential toxins.

### Response of detoxification genes when feeding on wildtype and mutant tomato

**Fig 5:**
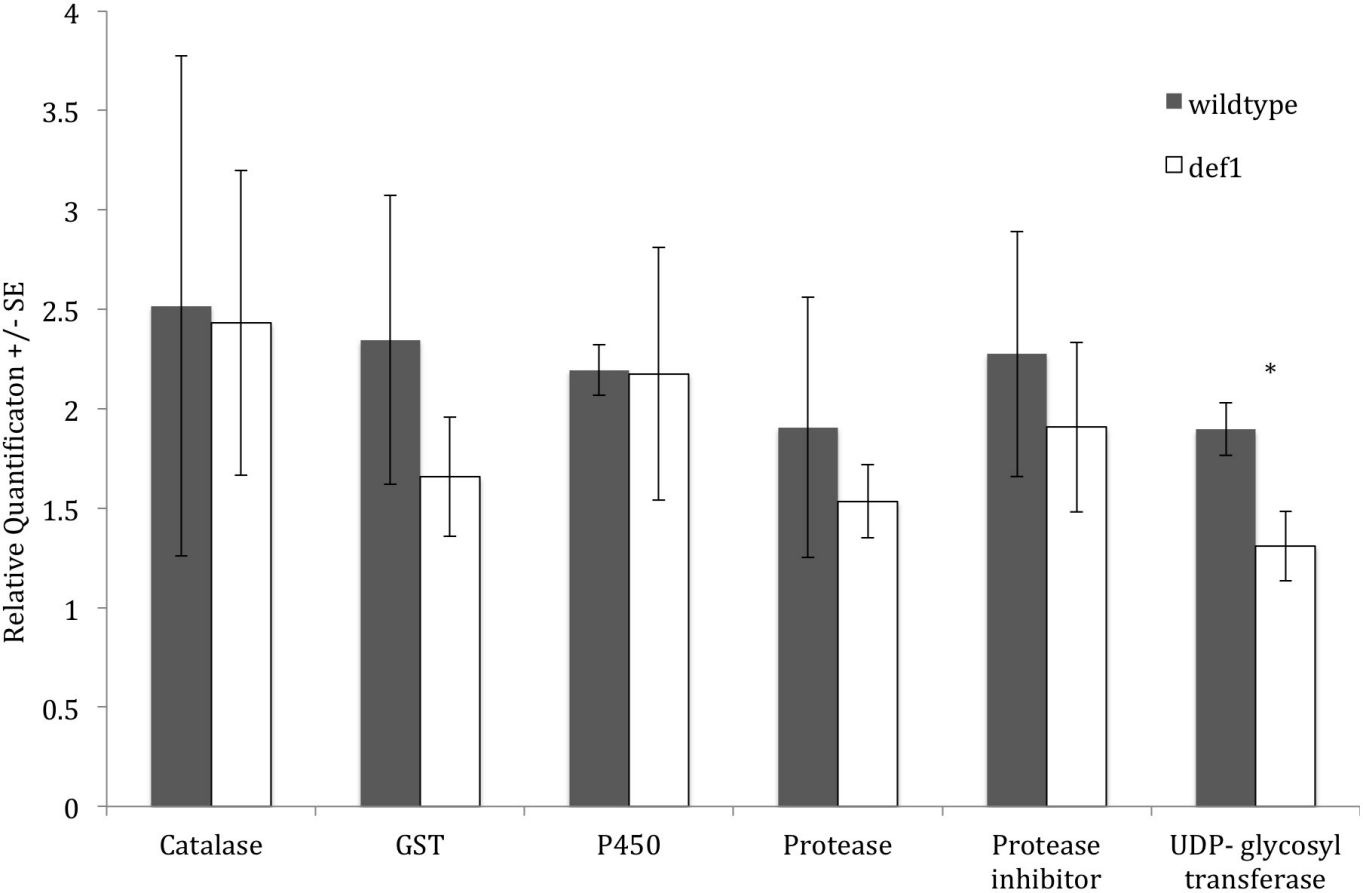
Gene expression of detoxification genes in salivary glands of cabbage looper feeding on wildtype and mutant (def-1) tomatoes. Salivary glands were dissected from 5^th^ instar larvae fed on wildtype or mutant tomatoes and used for real time qPCR analyses. Grey and white bars indicate expression of detoxification genes on salivary glands of larvae fed on wildtype and mutant tomatoes, respectively. Bars indicate standard error. Differences were analyzed using the nonparametric test Mann-Whitney U test. * indicates significance at p<0.05.

More differences in expression of detoxification genes were observed in the salivary glands of larvae reared on tomato (Fig 3). To confirm the induction of detoxification genes in salivary glands, cabbage looper larvae were reared on artificial diet and then transferred to def1 mutant and wildtype tomato. Def1 (Defenseless-1) tomato plants are mutants affected in the octadecanoid pathway and thus, upon herbivore attack, they do not induce wound-related defenses [39]. Comparing the salivary glands of caterpillars raised on wildtype and def1 mutant plants, we measured the gene expression of six detoxification genes identified in the RNASeq data as upregulated in tomato versus cabbage and/or artificial diet. The analyzed genes were a catalase, a glutathione-S-transferase, a cytochrome P450, a protease, a protease inhibitor and a UDP-glycosyl-transferase (Fig 5). UDP-glycosyl-transferase was the only gene significantly downregulated in larvae feeding on def-1 mutants compared to wildtype fed. However, there was a trend for higher expression in larvae feeding on wildtype plants for the rest of the genes. Based on this result, we can confirm that detoxification genes are expressed in the salivary glands of cabbage looper, but the expression differences observed in larvae fed on tomato plants versus artificial diets may be due to constitutive differences or the specific defenses associated with the host plants rather than differences in the induced defenses regulated by the octadecanoid pathway. In other words, differences in expression of detoxification genes in the salivary glands will vary more depending on whether the insect encounters a specific type of defense rather than the level of said defense. Also, because higher gene expression does not always translate to higher enzymatic activity [91], future studies assessing changes in enzymatic activity of all these enzymes could provide further evidence of their role in response to plant defenses.

### Cabbage looper saliva is plastic and potentially aids in detoxification of plant defensive compounds

In conclusion, transcriptome of the salivary glands of the generalist *Trichoplusia ni*, is plastic. First, diet or host plant has a significant effect on the expression of salivary gland genes. Second, one of the significantly regulated pathways corresponded to the spliceosome pathway. Changes in this pathway may alter splicing patterns and generate diversity in protein activity and function. Furthermore, we found evidence of expression of digestion and detoxification genes, as well as immune related genes that could be allowing *Trichoplusia ni* to respond not only to differences in host plant, but also to the microbiome associated with the host plants. The availability of this transcriptome library for the cabbage looper salivary glands opens the door to a myriad of research opportunities including using this information for functional studies (i.e. RNAi, CRISPR-Cas9) of specific salivary genes. Using these genomic tools will help elucidate the proximate and ultimate mechanisms that allow generalist insects to establish on a range of hosts.

## Supporting information

S1 Fig Cluster dendogram of biological samples. (TIF)

S2 Fig Validations of RNASeq results using real time qPCR. (TIF)

S1 Table Real time quantitative PCR primers. (DOCX)

S2 Table Trinity assembly statistics. (DOCX)

S3 Table Transcriptome IDs. (XLSX)

S4 Table Differentially expressed genes. (XLSX)
