# Supplementary figures and images for: Host plant driven transcriptome plasticity in the salivary glands of the cabbage looper (Trichoplusia ni)
Source: PLoS One. 2017 Aug 8;12(8):e0182636. doi: 10.1371/journal.pone.0182636 (PMC5549731; doi:10.1371/journal.pone.0182636)

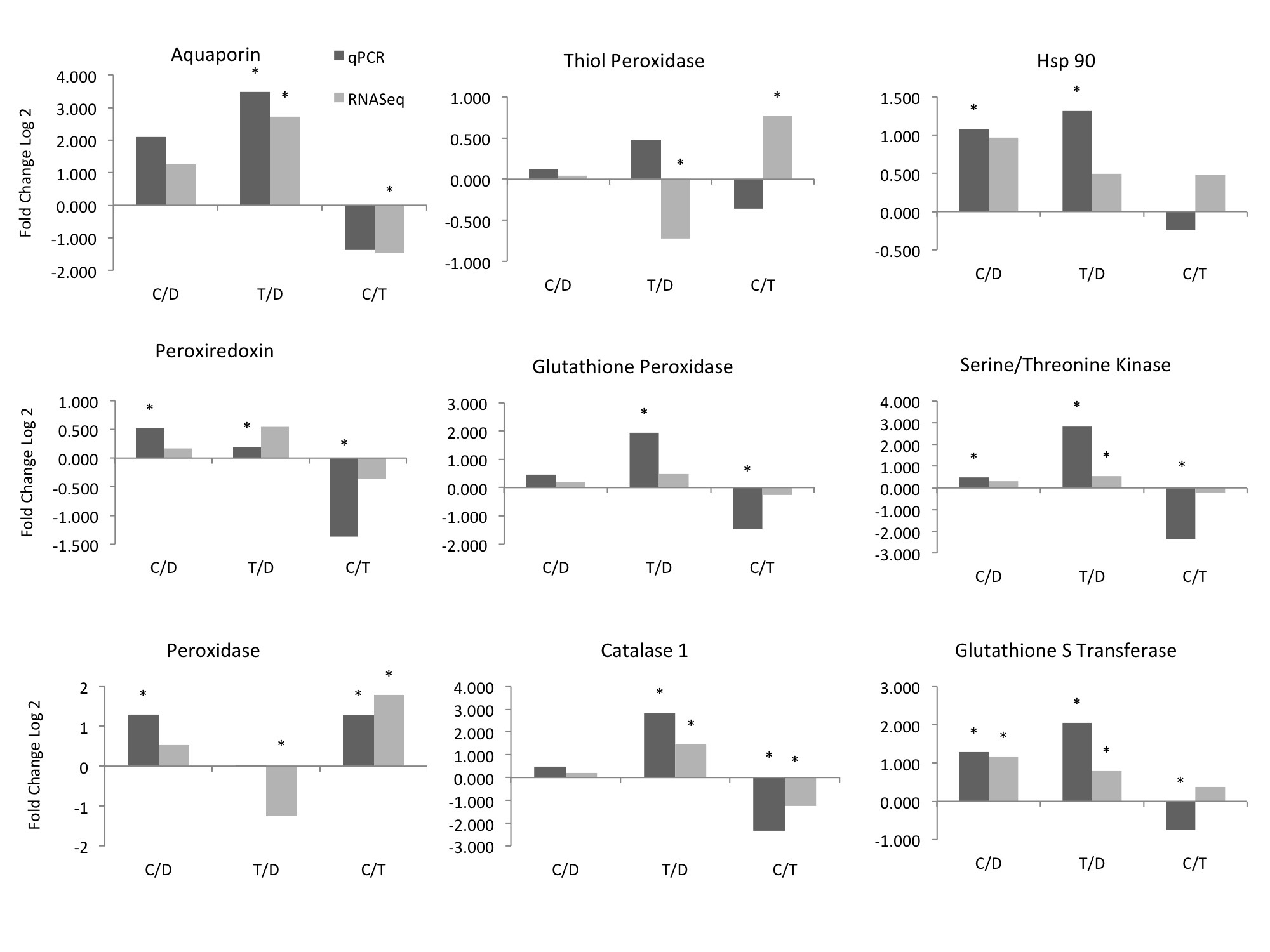

Supplement: S1 Fig — (TIF) [file pone.0182636.s001.tif]

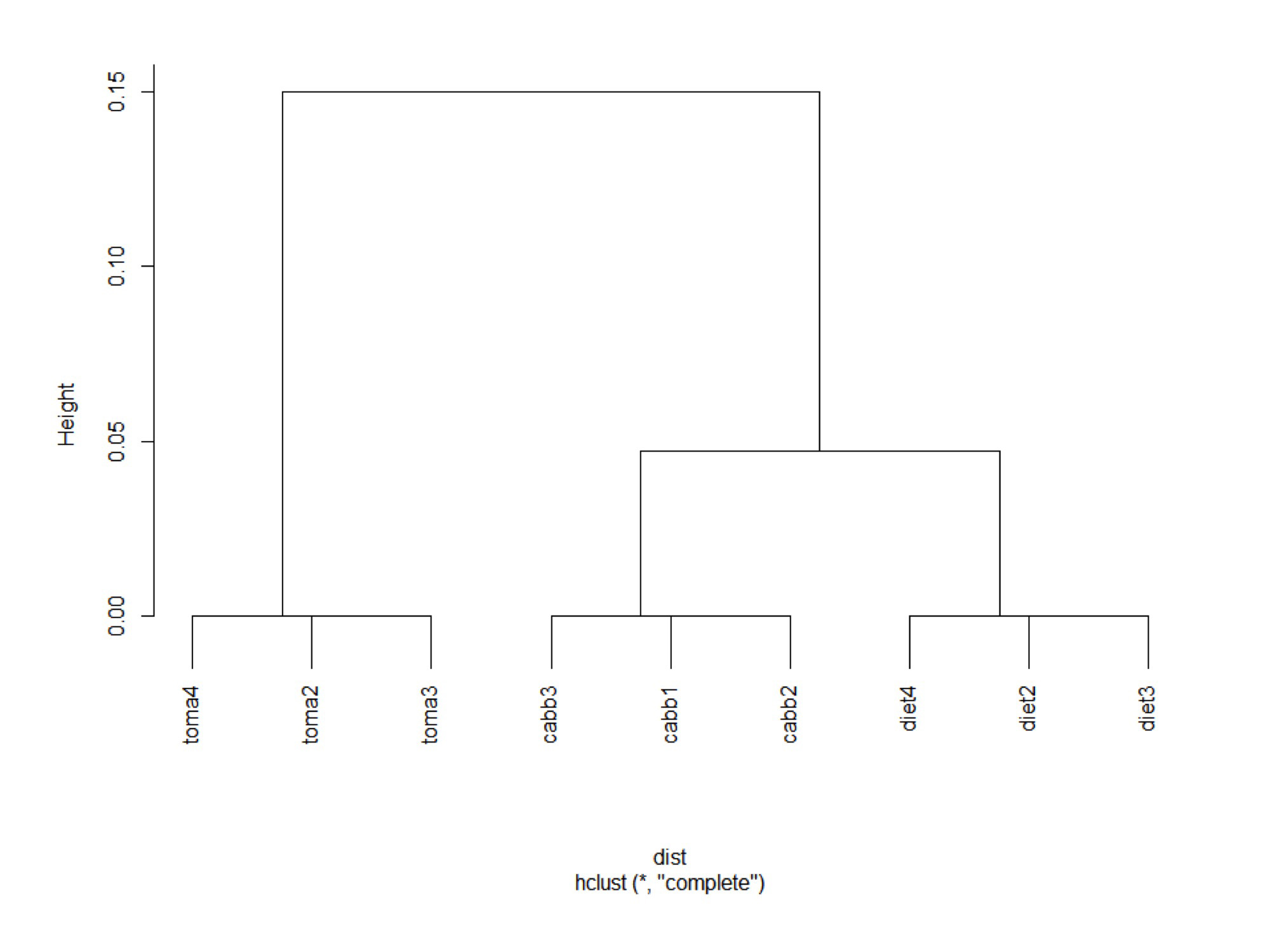

Supplement: S2 Fig — (TIF) [file pone.0182636.s002.tif]
