# Supplementary material for: Host plant driven transcriptome plasticity in the salivary glands of the cabbage looper (Trichoplusia ni)
Source: PLoS One. 2017 Aug 8;12(8):e0182636. doi: 10.1371/journal.pone.0182636 (PMC5549731; doi:10.1371/journal.pone.0182636)
Supplement: S1 Table — (DOCX) [file pone.0182636.s003.docx]

| **ID** | **Contig** | **Primer name** | **R-squared** | **Efficiency (%)** | **Sequence 5'-3'** |
| --- | --- | --- | --- | --- | --- |
| Aquaporin | comp155308 | TnAqp1 | 0.99 | 82 | F-GTCAGCCCATTCGTTAGC |
|  |  |  |  |  | R-CATCGAATTCTTCCTTGGATTTG |
| Thiol peroxidase | comp6532 | TnThPx1 | 0.98 | 100 | F-GTGTCGGCATTGGTTTCT |
|  |  |  |  |  | R-CTCCTTGATGAGGGCTTTG |
| Peroxidase | comp12985 | TnPer2 | 0.98 | 97 | F-GACCCGGAGAGAGTAGAAAT |
|  |  |  |  |  | R-GGGTCTGTTAGGTCTCTCATA |
| Hsp90 | comp5870 | TnHsp1 | 0.97 | 87 | F-GATACAACCACCTTCTCAACC |
|  |  |  |  |  | R-CGAGCCCATTGATGAGTATG |
| Peroxiredoxin | comp14252 | TnPXR3 | 0.98 | 84 | F-CTTCTACCCGATGGACTTTAC |
|  |  |  |  |  | R-GTGAGACTTGTCGCTGATG |
| Glutathione Peroxidase | comp15858 | TnGPX1 | 0.99 | 100 | F-ACGGCTGGACAGTTAAATC |
|  |  |  |  |  | R-GGATGTCCTTACACCATTCC |
| Serine/threonine kinase | comp11663 | TnSerkin2 | 0.97 | 100 | F-GCATCTTGCCTTGTCTATCA |
|  |  |  |  |  | R-TGGTACTCCAGTTCCGTATAA |
| **Catalase** | comp27660 | TnCat1 | 0.99 | 91 | F-GAAGTCACCCTTGGCAATAG |
|  |  |  |  |  | R-CTGATGGTTACAGACACATGAA |
| **Glutathione-S-transferase** | comp15433 | TnGST1 | 0.99 | 94 | F-CAAGAAGCAGGAGACCTTAG |
|  |  |  |  |  | R-GAGTAGACGTTGTCGATGAG |
| **Protease** | comp8423 | TniProt | 0.99 | 90 | F-CTCGATCCCTCGTGAGAATA |
|  |  |  |  |  | R-CCTACCGCCAGGACTATTA |
| **Proteinase inhibitor** | comp877 | TniPI | 0.98 | 100 | F-ACAAACCCTTCCTGTTCTTC |
|  |  |  |  |  | R-AACGCCATCTATCGCTTATAC |
| **Cytochrome P450** | comp12543 | TniP450 | 0.98 | 99 | F-GAACCGTGCCCGTTATTT |
|  |  |  |  |  | R-CTGGAACTCCTTCGAGTAAAC |
| **UDP-glycosyl transferase** | comp11498 | TniGlyT | 0.98 | 84 | F-CGTTAGATCGCAACCACTAC |
|  |  |  |  |  | R-GCGACACTTCATTCACTATCA |
| GAPDH | CF259232.1 | TniGAPDH1 | 0.99 | 98 | F-GCCAAGAAGGTCATCATCTC  R- GGTCATCAAACCTTCAACAATC |

Genes in bold were used for detoxification experiments
