# Supplementary material for: Host plant driven transcriptome plasticity in the salivary glands of the cabbage looper (Trichoplusia ni)
Source: PLoS One. 2017 Aug 8;12(8):e0182636. doi: 10.1371/journal.pone.0182636 (PMC5549731; doi:10.1371/journal.pone.0182636)
Supplement: S2 Table — (DOCX) [file pone.0182636.s004.docx]

| Total Trinity 'genes' | 30,082 |  |
| --- | --- | --- |
| Total Trinity transcripts | 38,649 |  |
| Percent GC | 41.38 |  |
|  |  |  |
|  | All transcripts | Longest isoform/gene |
| Contig N10 | 5,969 | 5,163 |
| Contig N20 | 4,604 | 3,900 |
| Contig N30 | 3,684 | 3,068 |
| Contig N40 | 2,999 | 2,422 |
| Contig N50 | 2,418 | 1,903 |
|  |  |  |
| Median contig length | 583 | 451 |
| Average contig length | 1,218.82 | 966.31 |
| Total assembled bases | 47,106,062 | 29,068,458 |
